# Developments in Alloplastic Bone Grafts and Barrier Membrane Biomaterials for Periodontal Guided Tissue and Bone Regeneration Therapy

**DOI:** 10.3390/ijms25147746

**Published:** 2024-07-15

**Authors:** Rabia Ashfaq, Anita Kovács, Szilvia Berkó, Mária Budai-Szűcs

**Affiliations:** Institute of Pharmaceutical Technology and Regulatory Affairs, Faculty of Pharmacy, University of Szeged, Eötvös u. 6, H-6720 Szeged, Hungary; rabia.ashfaq@szte.hu (R.A.); gasparne.kovacs.anita@szte.hu (A.K.); berko.szilvia@szte.hu (S.B.)

**Keywords:** autografts, xenografts, allografts, synthetic biomaterials, guided bone regeneration, alloplastic, periodontitis, membrane barriers, guided tissue regeneration

## Abstract

Periodontitis is a serious form of oral gum inflammation with recession of gingival soft tissue, destruction of the periodontal ligament, and absorption of alveolar bone. Management of periodontal tissue and bone destruction, along with the restoration of functionality and structural integrity, is not possible with conventional clinical therapy alone. Guided bone and tissue regeneration therapy employs an occlusive biodegradable barrier membrane and graft biomaterials to guide the formation of alveolar bone and tissues for periodontal restoration and regeneration. Amongst several grafting approaches, alloplastic grafts/biomaterials, either derived from natural sources, synthesization, or a combination of both, offer a wide variety of resources tailored to multiple needs. Examining several pertinent scientific databases (Web of Science, Scopus, PubMed, MEDLINE, and Cochrane Library) provided the foundation to cover the literature on synthetic graft materials and membranes, devoted to achieving periodontal tissue and bone regeneration. This discussion proceeds by highlighting potential grafting and barrier biomaterials, their characteristics, efficiency, regenerative ability, therapy outcomes, and advancements in periodontal guided regeneration therapy. Marketed and standardized quality products made of grafts and membrane biomaterials have been documented in this work. Conclusively, this paper illustrates the challenges, risk factors, and combination of biomaterials and drug delivery systems with which to reconstruct the hierarchical periodontium.

## 1. Introduction

The term periodontal refers to the area around the tooth, whereas periodontitis is a severe bacterial infection that causes damage to the gums and surrounding tissues in the mouth. If left untreated, the infection progresses, deteriorating the underlying bone around the teeth and leading to ultimate tooth loss. Surprisingly, tooth loss is not a painful process, which means that one may not even realize what is happening in the oral cavity [1].

It is believed that chronic periodontitis, which affects the supporting tissues of the teeth, may also increase the risk of systemic complications. The poor natural regeneration ability of damaged periodontal tissues and the constant presence of harmful microbes in the oral cavity highlight the need for effective clinical procedures to grow healthy periodontal tissues.

Periodontal regeneration refers to the process of restoring lost or damaged tissue with the aim of establishing new cementum and periodontal ligament fiber (PDL) (connecting cementum to the alveolar bone), and encouraging new bone formation. Several approaches and modalities are used in practice, such as guided bone and tissue regeneration, root surface modification, and growth factor delivery, to regenerate or repair periodontal damage [2].

The concepts of guided bone regeneration (GBR) and guided tissue regeneration (GTR) suggest that damaged tissues may benefit from the application of cells with regenerative properties, thus facilitating the repair and growth of the affected tissues. This idea has been successfully demonstrated and validated through numerous clinical trials [3].

The GBR technique is often used to replace damaged alveolar ridges and grow hard tissues when a dental implant is placed at the tooth extraction site. It also involves using a membrane to treat intrabony anomalies and furcations, promote bone growth, and protect the bone defect area from fibrous tissue infiltration. The permeable membranes used in GBR have the potential to facilitate the formation of healthy bone structures around damaged areas. Furthermore, GBR membranes can help repair bone structure after tooth extraction or loss and preserve the socket region [4].

GTR is a dental procedure that involves rebuilding periodontal and connective tissues, ligament, cementum, and lost gums around a tooth by separating gingival (epithelial) from alveolar bone/PDL tissue using an occlusive barrier membrane. Hence, during the procedure, a membrane is applied to the surgical site to prevent connective and epithelial tissue from migrating through it. This allows progenitor cells from the blood to reach the alveolar bone or the lining of the remnant periodontal ligament to recolonize the root area and divide to produce new periodontal supporting components [5].

Ideal barrier membranes should provide easy access to the site of interest, have a more rigid framework for space maintenance, and be porous, degradable, stable, and biocompatible. Therefore, using a (resorbable or non-resorbable) combination of grafts and membranes is beneficial in that it subsides epithelial down-growth, prevents barrier collapse, and maintains adequate space [6].

To encourage bone and tissue regeneration as well as accelerate the healing of bone deformities or fractures, a piece of bone or bone substitute is transplanted into a designated place during a bone graft treatment. The graft material can be obtained from the patient’s own body (autograft), from another individual of the same species (allograft), from different species or animal-derived sources (xenograft), or from synthetic biomaterial (alloplast). Bone grafts must meet certain criteria to ensure optimal performance. These criteria include an abundant supply of bone without harming the donor area, the ability to stimulate bone growth, no negative reaction from the host’s immune system, rapid revascularization, the ability to promote bone formation and encourage bone growth, minimal morbidity, and complete replacement of the graft with bone that resembles the composition of the host bone in both quantity and quality [7].

Three distinct bone growth characteristics of bone grafts are osteogenesis, osteoinduction, and osteoconduction. Osteogenesis is one of the three main bone healing mechanisms that refer to the formation of new bone by viable bone cells (osteoblasts or osteoprogenitor) within the graft material, generating new bone tissue by creating, depositing, and gradually mineralizing the bone matrix [8]. However, it is worth mentioning that not every bone graft material possesses osteogenic qualities for bone healing and treatment; osteoconduction and osteoinduction play critical roles in bone regeneration and healing roles otherwise.

The capacity of the graft material to cause the host cells to develop into osteoblasts (to produce bones) is referred to as osteoinduction. Osteoinduction encourages host cells to develop into bone-forming cells. Bone morphogenetic protein (BMP) is a well-known naturally occurring osteoinductive growth factor and is considered important for regulating bone development and healing by stimulating the differentiation of mesenchymal stem cells into osteoblasts [9].

In addition to BMP, transforming growth factor-beta (TGF-β) and insulin-like growth factor (IGF) present in naturally occurring proteins, recombinant proteins, or platelet-derived growth factors (PDGFs) also affect the bone healing process [10].

Osteoconduction provides a template for bone growth during the bone regeneration process. The graft material acts as a scaffold, guiding the migration of osteoblasts from the host bone into the graft and facilitating the attachment of host bone cells to the graft. Because of the porous nature of graft materials, blood vessels and host cells can penetrate into and integrate with grafts to generate bones [11].

However, natural grafts come with associated hazards and risks, including additional time-consuming surgeries, postoperative pain, infection or fracture at the donor site, quality variation within harvested bones or tissues, high morbidity, compromised dimensional stability, increased costs, and religious/ethical controversies (in the case of xenografts) [12].

Thus, the growing demand for materials has sparked interest in investigating various biomaterials to address issues, such as the limited supply of autogenous and allogeneic bone, risks of disease transmission, and the porosity of xenogeneic bone particles [13].

Synthetic materials have presented superior biological behavior in the process of bone formation that have been further confirmed through in vivo experiments conducted on mice, minipigs, and humans.

Although alloplastic graft materials lack the ability for osteogenesis and osteoinduction, they possess that for osteoconduction, which encourages the growth of new bone with low morbidity. They also offer better dimensional stability and sufficient material supply with volume maintenance capacity, permitting cell infiltration and remodeling with limited or no risk of infection [14]. Thus, commercially available alternatives for bone grafting aim to attain a similar osseoregenerative profile to that of natural grafts. A summarized overview of the classification and examples of all possible types of bone grafts, their substitutes, specific features, and commercially available products are provided in Table 1.

Scientists have explored the use of synthetic alternatives like composite grafts, polymers, and inorganic materials to restore osseous tissues. For example, the polylactide-coated TCP (tricalcium phosphate) synthetic graft was proven to be effective in repairing bone structure in rabbits while maintaining biocompatibility [15].

Calcium phosphate ceramics enable the adhesion, proliferation, and growth of host bone cells. Over time, these grafts may be resorbed or result in the healing of a bone defect or fracture [16].

Barrier membranes and/or growth factors are frequently added to alloplastic bone substitutes to overcome challenges related to the regeneration process [17]. Barrier membranes (both biodegradable and non-biodegradable) are implanted to enhance stability, volume maintenance, and prevent unwanted cell infiltration from the gingival epithelium and connective tissue. Ideally, this membrane protects periodontal tissue regeneration for up to 6 weeks and provides a 24-week shielding effect for bone augmentation therapy, thus providing the necessary space for tissue regeneration, guiding periodontal ligament cells, and bone regeneration [18].

This review will briefly describe natural graft types and provide a detailed overview of synthetic replacement options (alloplasts), along with barrier membranes. This paper is aimed at revealing how to regenerate the hierarchical and functional periodontium, serving as a guide for future advancement in this field.

**Table 1:** Available natural grafts from commercial tissue banks, marketed synthetic biomaterial products, and their features.

Main Classes	Graft-Type Examples	Source	Histology/ Bone Source	Bone Graft Form	General Features	Commercial Products	Ref.
Autografts	Intraoral: incisive fossa, coronoid process, zygomatic body, anterior maxillary sinus wall, nasal spine, ascending ramus, maxillary tuberosity, mandibular symphysis, palate, torus Extraoral: iliac crest, cranium, radius, tibia, rib, fibula	Patients themselves	Cortical, cancellous, corticocancellous	Blocks	Bearing osteoinductive, osteoconductive, and osteogenic potential, with limited graft volume, absence of immunogenic reaction, and disease transmission	-	[19]
Allografts	Freeze-dried bone, demineralized freeze-dried bone, fresh and/or frozen bone	Different donors of the same species or genetically non-identical members	Cortical, cancellous, corticocancellous, osteoarticular	Particulate bone	Bearing osteoinductive and osteoconductive potential; availability in large amounts with various particle sizes	MTF^®^-FDBA (Musculoskeletal Transplant Foundation: Edison, NJ, USA), OsteoSponge (Xtant medical: Belgrade, MT, USA), DynaBlast^®^ (Keystone Dental Group: Burlington, MA, USA), Puros^®^ (Zimmer Biomet: Warsaw, IN, USA), MinerOss (BioHorizons: Birmingham, AL, USA), Dynagraft^®^ (Keystone Dental Group: Burlington, MA, USA), Grafton^®^ (BioHorizons: Birmingham, AL, USA), MTF^®^-DFDBA (Musculoskeletal Transplant Foundation, Edison, NJ, USA), Raptos^®^ (Citagenix: Laval, QC, Canada), DBX^®^ Putty (Dentsply Sirona Inc., Charlotte, NC, USA), Opteform^®^ (Exatech Inc.: Gainesville, FL, USA)	[20]
Xenografts	Bovine Hydroxyapatite, Coralline calcium carbonate, Porcine bone, Equine bone, Algae	Grafts from different species such as cats, dogs, rats, cows, bullfrogs, sheep, pigs, and chickens.	Cortical, cancellous, corticocancellous	Slurry	Osteoconductive, bone minerals with no or fewer organic elements, high availability, limited resorptive potential	FRIOS^®^Algipore^®^ (Dentsply Sirona Inc., Charlotte, NC, USA), Bio-Oss^®^ (Geistlich Pharma: Wolhusen, Switzerland), Cerabone^®^ (Botiss biomaterials GmbH: Zossen, Germany), Gen-OS^®^ (Tecnoss dental: Torino, Italy), Biocoral^®^ (Inoteb, Saint-Gonnery, France), Osteobiol^®^ (Tecnoss dental: Torino, Italy), Pro Osteon^®^ (Zimmer Biomet: Warsaw, IN, USA), Interpore-200^®^ (Interpore International: Irvine, CA, USA), OsteoGraf/N (Dentsply Sirona Inc.: Charlotte, NC, USA), MinerOss^TM^ X (BioHorizons: Birmingham, AL, USA)	[21]
Alloplasts	Calcium phosphate ceramic Hydroxyapatite Tricalcium phosphate Biphasic calcium phosphate cement Calcium phosphate cement Calcium sulfate Bioactive glass Polymers	Synthetic biomaterials	-	Powder, paste	Osteoconductive, bears similarity to bone mineral, degradable and non-degradable nature, low cost	Biogran^®^ (Biomet 3i Innovations Inc.: Plam beach gardens, FL, USA), BonePlast^®^ (Zimmer Biomet: Warsaw, IN, USA), Cortoss^®^ (Stryker Corporation: Kalamazoo, MI, USA), Guidor^®^ easy graft (Sunstar: Osaka, Japan), Hydroset^®^, IngeniOs^®^ (Zimmer Biomet: Warsaw, IN, USA), B-Ostin^®^ (Basic Healthcare: Himachal Pradesh, India), Perioglass^®^ (Novabone: Jacksonville, FL, USA), Rhakoss^®^ (Orthovita, Inc: Malvern, PA, USA), Vitoss^®^ (Stryker Corporation: Kalamazoo, MI, USA)	[22]

## 2. Search Methodology

A rigorous and systematic literature search was conducted to identify and evaluate the available data on relevant topics from several scientific databases such as Web of Science, Scopus, PubMed, MEDLINE, and Cochrane Library for entries published up to 2024. The objective was to be comprehensive, critical, and transparent in our search methodology. We designed our search strategy with a focus on several important terms and phrases, combined with Boolean operators. The keywords “natural grafts”, “synthetic grafts”, “synthetic biomaterials/alloplastic”, “barrier membranes”, “guided bone regeneration”, “guided tissue regeneration”, “periodontal regeneration” and “available brands/ marketed products” were thoroughly searched for. Clear inclusion/exclusion criteria for assessing the quality of the studies were established during the screening process. This helped to distinguish high-quality evidence. Case reports, series, and review articles without original data, biomechanical studies without clinical outcomes, and non-biocompatible materials were the exclusion criteria, while randomized controlled trials, prospective cohort studies, and retrospective studies comparing different graft types, articles only concentrating on periodontal regeneration and written in the English language, and papers published in last 15 years were inclusion criteria.

## 3. Brief Introduction to Natural Bone Substitutes

### 3.1. Autografts

A patient’s own bone is used in an autograft, also known as an autologous or autogenous bone graft.

Autogenous bone grafts from both extraoral and intraoral sources have been utilized in periodontal therapy owing to their osteogenic potential. Several case reports have demonstrated successful bone fill in dehiscences, furcations, and intraosseous sites using autogenous iliac cancellous bone with marrow [19].

According to an extensive series of case reports, intraosseous defects showed a mean bone fill ranging from 3.3 to 3.6 mm, while a 2.5 mm increase in crestal bone height was observed. Histological evaluation of treated sites showed supra-crestal bone apposition and limited periodontal regeneration, as suggested by a reference notch placed at the alveolar crest [23].

A mean bone fill of 3.4 mm was reported, which filled more than 50% of the initial defect [24]. Another controlled study indicated a modest bone fill of 1 mm in defects treated with intraoral grafts [25].

### 3.2. Allografts

In general, three types of bone allografts are available in commercial tissue banks, including fresh, frozen, freeze-dried bone allografts (FDBA), and decalcified freeze-dried bone allografts that have been extensively field-tested and are commonly used in adult periodontal therapy [20].

FDBA has several advantages over autografts. Obtaining enough autogenous bone from an intraoral location for treating multiple defect sites can be challenging, and additional surgical procedures are often required, which may result in higher postoperative problems such as root resorption and exfoliation–sequestration. Moreover, rapid reappearance of defects is associated with autogenous bone grafts, which has not been reported with paniculate freeze-dried bone allografts [26].

Controlled clinical trials for the treatment of periodontal defects have shown that the use of FDBA can result in bone fills ranging from 1.3 to 2.6 mm. FDBA can be obtained in large quantities from tissue banks and has an indefinite shelf life due to vacuum storage conditions [27].

In total, 997 bone defects were implanted by 89 clinicians using freeze-dried bone allografts, while 524 periodontal bone defects were treated with a combination of freeze-dried bone allografts and autogenous bone. A one-year follow-up radiographic evaluation provided sufficient data from 381 sites treated with allografts alone and 176 sites treated with allografts plus autogenous bone to determine the effectiveness of the surgical treatments. Results showed recovery in periodontal osseous defects in 67% of sites treated with freeze-dried bone alone and 78% of sites treated with combination grafts. Additionally, probe depth reduction was up to 10% greater with combined therapy. Hence, it was concluded that composite bone grafts have more potential to treat furcation defects, two-wall defects, pocket reduction, and osseous regeneration [28].

The concomitant use of wound closure flaps and antibiotics was found to enhance graft success, as documented in surgical re-entry. Mabry and colleagues randomly assigned juvenile periodontal patients into two groups: one treated with FDBA alone and the other treated with FDBA combined with tetracycline. Reentry data collected from 104 different defect sites demonstrated promising bone fill and bone defect resolutions with the combination of tetracycline and FDBA (4:1). This combination was found to be the treatment of choice for localized juvenile periodontitis compared with FDBA alone [29].

Another type of allograft material used in oral and maxillofacial surgery is the decalcified freeze-dried bone allograft (DFDBA), which possesses both osteoinductive and osteoconductive qualities. This material is obtained from human bone tissues that undergo a rigorous screening process to ensure safety. The bone tissue is decalcified to remove inorganic minerals, thereby eliminating potential risks such as immunogenicity and disease transmission. Following decalcification, the bone tissue is frozen at very low temperatures to maintain its biological qualities [30].

In many dental and maxillofacial operations, including sinus lifting, ridge augmentation, and periodontal defect repair, DFDBA is a great option for encouraging bone regeneration and integration with natural bone. DFDBA has been tested on human subjects and has shown bone fill results similar to those of FDBA, ranging from 1.7 to 2.9 mm. A systematic review revealed consistently better results in bone fill with DFDBA compared with that achieved with open-flap debridement procedures [31].

Calvarial defects in 35 guinea pigs were treated with graft materials, and the osteogenic potential of cortical DFDBA was compared with that of autogenous bone, osseous coagulum, and FDBA. It was determined via histology and radionuclide uptake analysis that autogenous materials and osseous coagulum have low osteogenic potential in this model system, while DFDBA possesses significant osteogenic activity. This clinical study suggests that the decalcified freeze-dried bone allograft may be a better graft for dental and therapeutic applications [32]. Table 2 outlines the subtypes of all graft options, their mechanisms, merits, demerits, and commercial products.

### 3.3. Xenografts

Bovine-origin bone replacements are an example of widely available xenografts in the commercial market (like BioOss^TM ^ Geistlich-Pharma, Wolhunsen, Switzerland) and are the most well-documented materials in this class. They are osteoconductive, and after lyophilization and deproteinized treatment, they stop triggering an immunological reaction [56].

However, instead of going through the typical bone remodeling process, the granules of these materials are thought to absorb poorly or slowly. The possible reason for their reduced absorption potential is due to the modification of the structure of hydroxyapatite as a result of high-temperature processing applied to prevent immunological reactions, allergies, and infectious disorders [57].

Moreover, it has been shown that bone replacements produced from equines can stimulate angiogenesis and osteoblastic development while being absorbed by osteoclasts. Furthermore, a six-month postoperative evaluation confirmed the presence of neoplastic bone related to remodeling effects around the graft material with successful sinus lift instances [58].

Given the similarities between human and pig genomes, osteoconductive porcine-derived replacements have recently been developed. Although they come with a low risk of disease transmission, they lead to inadequate development of neovascularisation and decreased absorption ability over time [59].

Antigenicity and inflammatory host responses are absent in algal bone derivatives. This is why this biomaterial has been mixed with different growth factors such as BMPs and TGFβ1, resulting in successful sinus expansion by increasing cancellous bone around biomaterials and progressively replacing it with newly produced bone [60].

There are striking parallels between macroporic corals (Porites, Pocillopora, Lobophyllia, Acropora, Polyphillia, and Goniopora) and cancellous bone. After sterilization and protein treatments, corals are used as bone graft substitutes (Biocoral). Coral grafts have been used to treat jaw abnormalities, facilitate cell attachment, and enhance bone development ability due to their osteoconductive qualities and ability to carry growth factors [61].

## 4. Alloplastic Bone Graft Biomaterials

Alloplastic grafts, or synthetic bone grafts, are cost-effective and osteoconductive options that allow practitioners to avoid the drawbacks associated with natural bone grafts. Common alloplastic grafts used in regenerative dentistry include non-porous hydroxyapatite (HA), hydroxyapatite cement, porous hydroxyapatite, beta-tricalcium phosphate (β-TCP), bioactive glass, and PMMA and HEMA polymers (calcium-layered polymers of polymethylmethacrylate and hydroxyethylmethacrylate). It has been reported that porous and non-porous HA materials, as well as PMMA and HEMA polymers, are less or non-resorbable, while tricalcium phosphate and bioactive glass are bioabsorbable. Among these, β-TCP is a widely used biocompatible option with vascular and cellular growth capacity and an acceptable disintegration rate [22].

According to a meta-analysis and extensive research, β-TCP may provide additional clinical benefits for treating attachment levels and periodontal pocket depths in infrabony defects [62].

### 4.1. Calcium Phosphate Ceramics

Synthetic mineral salts or extremely crystalline materials are sintered at temperatures exceeding 1000 °C to create calcium phosphate (CaP) ceramics. These ceramics have the same clinical and chemical composition as that of tooth and bone minerals, making them resorbable and biocompatible [21].

CaP ceramics with several key features have been found to be suitable for medical applications, including bioactivity, biocompatibility, bioactive fixation, biostability, controlled crystallinity, interfacial stability, osseointegration, osteoconductivity, osteoinductivity, and therapeutic capabilities. Their ability to partially dissolve and release ionic compounds to promote proliferation, differentiation, and cell adhesion is the primary mechanism underlying their remarkable bioactivity [63]. 

CaP ceramics are primarily used in dentistry for the following purposes: replacing teeth, augmenting alveolar bone, sinus lifts, repairing large bone defects caused by tumors, scaffolds for dentin regeneration, and coatings on titanium alloy implants to combine the strength of the metal with the bioactivity of the CaP ceramics [64].

Ceramic materials have two main drawbacks: they are difficult to mold into the correct shape and have low mechanical strength. Examples of calcium phosphate ceramics include hydroxyapatite, biphasic calcium phosphate, and tricalcium phosphate [37].

A clinical trial was conducted to assess the effectiveness of biphasic calcium phosphate ceramic in treating periodontal osseous abnormalities; 137 veteran patients were divided into three treatment groups for three three-year studies. Both autogenous bone implants and open-flap curettage techniques were evaluated against each treatment group. For every patient, the baseline probing attachment level, Navy plaque index, and gingival index were noted. By the end of the trial period, 101 patients had completed the trial. Despite a gradual increase over time in the gingival and plaque indices, no statistically significant differences were seen between all three therapy groups. Subjects in the curettage group showed a gain in the attachment level of 0.9 mm, those in the ceramic group showed a gain of 1.0 mm, and those in the bone implant group showed a gain of 0.4 mm. Despite them having a higher gain, biphasic calcium phosphate ceramic patients’ differences were not statistically significant [65].

#### 4.1.1. Hydroxyapatite

Hydroxyapatite (HA) is a biocompatible bone graft material with a composition similar to that of natural bone. It is osteoconductive and can be osteoinductive under certain conditions. Although HP has high tensile strength and compression, its slow resorption rate can impede rapid recovery and bone remodeling, as it may remain years after implantation. However, the nanocarriers of HP present an alternative to overcoming the slow resorption issue when there is a small size and large surface area. Nanocrystalline HA (n-HA) is easy to produce at lower sintering temperatures [66].

By comparing their chemical formulae, the inorganic component of the bone matrix and HA share an almost identical composition. HA, with its promising characteristics (such as osseointegration, osteoconduction, and osteoinduction), is used for various medical purposes, such as bone grafts, dental implants, and coatings. However, its high brittleness, crystallinity, Ca/P ratio, low mechanical strength, and weak resistance to fracture have been barriers to achieving a higher HA resorption rate [67].

The field of nanotechnology has opened up new possibilities for nano-bone graft formation. Significant attention is being paid to n-HA crystals, which not only produce a less inflammatory reaction in comparison to porous HA but also exhibit good biocompatibility, improved resorption, and bioactivity to aid in new bone growth. Therefore, n-HA-based nanocomposites could provide better and faster healing substitutes in dentistry [68].

#### 4.1.2. Tricalcium Phosphate (β-TCP)

Tricalcium phosphate is regarded as the “gold standard” for synthetic bone grafts because its chemical composition closely resembles that of human bone [37].

Tricalcium phosphate is a substance meticulously researched for bone replacement in regenerative medicine because of its excellent biocompatibility, resorbability, and osteoconductivity. This material can be used to construct patient-specific resorbable implants with particular pore architectures and geometries based on their properties. A second surgical procedure to remove the implant is not required because these implants will be reabsorbed by the body during the healing process and replaced by natural bone tissue [69]. 

β-TCP typically gets resorbed within 3 to 5 months after implantation and has a mechanical strength similar to that of cancellous bone. However, it is not recommended for use in areas of the body that are subjected to mechanical loads due to its unpredictable degradation. It is most suitable for treating bone defects resulting from trauma or tumors [70].

The effects of PLGA-coated β-TCP (PLGA-β-TCP) on the preservation of the alveolar ridge were compared in a multicenter randomized controlled trial. The findings indicated that PLGA-β-TCP yielded comparable results in terms of implant survival and feasibility up to a year of post-loading, the stability of peri-implant bone level, and the maintenance of alveolar bone dimensions [71].

A prospective clinical trial conducted on ridge preservation demonstrated that the addition of HA and BCS (biphasic calcium sulfate) to β-TCP resulted in better healing in horizontal dimensional alterations than in spontaneous healing [72].

Recently, a meta-analysis’ findings, considering the limits of the included evidence, implied the possibility of clinical improvement in probing pocket depth and clinical attachment level with the use of a tricalcium phosphate material as compared with open-flap debridement in infrabony defects [62].

#### 4.1.3. Biphasic Calcium Phosphate

Biphasic calcium phosphate (BCP) is created by combining hydroxyapatite and tricalcium phosphate, which provides benefits from both of these substances. Adjusting the ratio of hydroxyapatite to tricalcium phosphate allows for variations in the duration of resorption and mechanical properties. The combination of mechanical stability and resorption time promotes bone growth and ensures the stability of the material. Adopting a biphasic calcium phosphate bone transplant can be advantageous for large defects and load-bearing areas due to the characteristics mentioned above [73].

Recently, a comparative study was conducted to assess the clinical and radiographic results of the treatment of peri-implant bone defects using demineralized bovine bone as a control and BCP (40% β-TCP and 60% HA) as a test. The results 18 weeks post-treatment showed good clinical, radiological, and wound healing outcomes that were in accordance with those of the control group [74].

Maxresorb^®^ (Botiss biomaterials GmbH: Zossen, Germany) is a 100% synthetic, biphasic bone substitute material composed of 60% HA and 40% β-TCP, designed to have a controlled resorption profile, with the HA component resorbing slowly and the β-TCP component resorbing faster to help maintain volume stability during bone regeneration. Bielenstein and colleagues recently conducted a study to analyze the in vivo responses of xenogeneic bone substitute materials (Cerabone^®^ Botiss biomaterials GmbH: Zossen, Germany) with biphasic calcium phosphate, a synthetic bone substitute (Maxresorb^®^). Comparable levels of bone regeneration were seen in both bone substitute materials groups. By using xenogeneic bone substitute materials as a control, histopathological scoring showed that the synthetic bone substitute displayed non-irritant behavior at all time points. Overall, the findings supported the hypothesis that Maxresorb^®^ is a good biocompatible natural bone substitute [75]. 

Similarly, another 6-month-long comparative study was performed with injectable biphasic calcium phosphate (Maxresorb^®^ inject) after socket preservation. The alveolar ridges of 21 patients were treated with Maxresorb^®^, which was injected into the alveolar ridges of patients in the test group, while patients of the control group were treated with bovine xenograft (Cerabone^®^). After 6 months, with the purpose of quantitative and qualitative histologic analysis, a re-entry procedure was carried out to collect bone biopsies. Results showed good osteoconductivity comparable to that in new bone development, complete biomaterial integration, biocompatibility, and an absence of an inflammatory tissue reaction within grafts and tissues in both groups [76]. 

These results were in accordance with those of another randomized controlled clinical study conducted on two groups receiving injectable biphasic calcium phosphate (Maxresorb^®^) and an organic bovine bone (Bio-Oss^®^: Geistlich Pharma, Wolhusen, Switzerland), where Maxresorb^®^ was found to be equally suitable for alveolar bone regeneration [77].

Recently, magnesium-doped biphasic calcium phosphate (Mg-BCP) has drawn interest as a potential substitute for bone. Mg-BCP is a blend of two Mg^2+^-doped bioceramics: β-TCP and HA. It has been found that, in comparison with the HA lattice, the β-TCP lattice displays preferential accommodation of Mg^2+^. The apatite forming capacity of BCP is observed to be enhanced by the addition of Mg^2+^, resulting in improved physicochemical features when compared with those of pure BCP. Based on in vitro results, Mg-BCP seems to be bioactive and cytocompatible. Mg-BCP’s biocompatibility and efficacy as a bone substitute were further validated and affirmed through in vivo studies [78]. 

SEM results demonstrated that BCP’s composition has been carefully chosen to offer a sufficient scaffold and good porosity for bone growth. SEM analysis also made it clear that BCP is a matrix made up of a macro-organized microstructure. Particle size distribution is homogeneous, with a center at a value slightly larger than the expected 500 μm, according to laser granulometry. XPS confirmed the presence of adventitious carbon on all sample surfaces and also demonstrated significant deviations from predictions in the Ca/P and O/Ca ratios in each sample [79].

### 4.2. Calcium Phosphate Cement

Calcium phosphate cement (CPC) is a mixture/paste made of liquid and powdered forms of calcium phosphate with or without API that fills and solidifies in defective sites at body temperature. Gradual absorption in the modeling process proves that CPC is a good osteoconductive material [80].

The strength of calcium phosphate cement itself is considerably lower than that of CaP ceramics. However, its self-setting capability and high biocompatibility make calcium phosphate cement a unique biomaterial. Among the unique benefits of calcium phosphate cement are its near-perfect cement adaptation to tissue surfaces in a defect and its ideal rate of resorption followed by the production of new bone. In its present state, CPC appears to be suitable for several applications. [81]. CPC is composed of calcium phosphate powders that harden at body temperature by forming a nanocrystalline structure with high porosity and a large surface area [82]. 

CaP ceramics, despite their high degree of hardness, have limited resorbability and a limited bone remodeling process which can be addressed by mixing hydroxyapatite with more biodegradable tricalcium phosphate (TCP) to form biphasic calcium phosphate (BCP). Sintering temperatures between 1000 and 1500 °C can eliminate hydroxide functional groups in the hydroxyapatite matrix due to dehydration, leading to the decomposition of hydroxyapatite into β-TCP and tetra-calcium phosphate (TTCP) [83]. Although CaP ceramics and cement both encourage bone regeneration, CPC with a larger surface area showed improvement in its biological activity.

In combination with Vicryl meshes or chitosan, CPC was found to improve mechanical strength [84]. Another study was carried out to address the poor mechanical characteristics of CPC and aimed to improve its toughness and flexural strength. Polyvinyl alcohol fibers were reinforced with CPC and examined through a variety of mechanical and biological test techniques that resulted in stabilized dental implants in fiber-reinforced CPC form [85].

### 4.3. Calcium Sulfate

Dehydrated calcium sulfate (plaster of Paris), first used as a synthetic biomaterial for bone graft in 1892, provides the fastest-dissolving synthetic option, breaking down within 1–3 months after implantation. However, the problem with the rapid disintegration of this graft poses a problem as it tends to resorb faster than natural bone [86].

Calcium phosphate grafts are preferred over calcium sulfate grafts because their porosity makes them more osteoconductive. However, calcium sulfate grafts are beneficial because of their low cost and ease of preparation. They should not be used in load-bearing areas as they lose their mechanical properties over time. Another issue with calcium sulfate grafts is the occurrence of serious wound drainage after implantation [37]. Hence, long-term studies are still needed to evaluate the effectiveness of calcium phosphate ceramics on periodontal regeneration.

### 4.4. Bioactive Glass

Bioactive glass, also known as “bioglass,” is a type of silica-based biomaterial that is used in artificial bone transplants. It differs from other synthetic bone grafts because it can chemically attach to both soft tissue and bone. For bioglass to be effective, the SiO_2_ content must be less than 60% of the weight of the glass. A bioglass with 45–52% SiO_2_ by weight will bind to both soft tissue and bone, while a bioglass with 55–60% SiO_2_ by weight will bind only to bones [87].

Bioactive glass, known for its ability to activate the body’s natural regeneration process, earns its name from this bioactive property. By activating genes responsible for bone formation, the bioactive glass begins to break down within 48 h after being implanted. Unlike manufactured hydroxyapatite, which can only bind to hard tissues, bioactive glass can link to both soft and hard tissues. Furthermore, bioglass has antibacterial properties due to its alkaline composition [50].

To address periodontal osseous anomalies, a comparative study (both clinically and radiographically) was performed with open-flap debridement, biphasic calcium phosphate (Ossifi^®^), and bioactive glass. In total, 35 patients with 45 administration sites were selected for this study from two test groups (test 1, bioactive glass; test 2, Ossifi^®^) and one control group (open-flap debridement). Pocket depth, clinical attachment level, and gingival and plaque index were assessed at baseline. Post-surgery baseline radiological features were calculated to obtain the percentage and amount of defect resolution. Subsequently, significant statistical differences were seen in all groups [88].

### 4.5. PMMA

Bone cement, polymethyl-methacrylate (PMMA), is a polymerized ester of acrylic acid, a clear stiff plastic with an exceptional compressive strength that has been used for many decades. In the early 1930s, Crawford and Rowland Hill commercially developed and used windscreens for airplanes and bubble canopies for gun turrets during World War II [89]. Later in the 1960s, PMMA was used as bone cement in hip replacement surgery [90].

PMMA was considered to be an inert and effective carrier for the delivery of antibiotics to infected bone voids. Buchholz and Engelbrecht introduced this concept to treat bone infections, as PMMA has the ability to release antibiotics and ions from its surface [91]. The PMMA preparation methods and intraoperative techniques determine the porosity of the graft materials. Manufacturers exploit these parameters to impact the clinical applicability of their products [92].

However, intraoperative variations and instability in resorption potential may cause problems in some cases. For example, in the case of PMMA-treated bone void where further bone reconstruction is required, removal of PMMA necessitates additional surgery.

When defects grafted with PMMA and HEMA polymer were compared with those in nongrafted controls, some notable improvements in clinical outcomes were observed. Although the effect of this particular category of bone grafts on therapy appeared to be significant, the effect varied between investigations [93].

Additionally, there is some histological evidence suggesting that very little regeneration might occur with polymer grafts made of PMMA and HEMA. Nevertheless, currently, it appears that alloplastic components serve as a filler that does not irritate the skin. Comparisons between alloplasts and bone allografts indicate comparable clinical outcomes. A recent systematic review study found that the clinical outcomes of bovine HA and particulate bone transplantation were similar [94].

The above-mentioned synthetic biomaterials used in bone grafting may be cytotoxic to varying degrees. Numerous cytotoxicity investigations were carried out on graft materials utilizing various cell lines. In a recent study, the cytotoxicity of synthetic and bovine-derived bone graft materials produced from cows was evaluated utilizing dental pulp stem cells and Saos-2 cells as an in vitro test bed. Results showed significant toxicity with both of the aforementioned graft materials, while in case of allograft bone materials, the dental pulp stem cell line did not show toxicity [95].

Similarly, another cytotoxicity study with polycaprolactone/collagen vascular grafts showed that synthetic vascular grafts can exhibit toxicity, but the cytotoxicity of their degradation products is generally limited. The cell viability of human umbilical vein endothelial cells on collagen vascular grafts was higher than that of collagen-free vascular grafts, which showed improved cell proliferation and graft biocompatibility with reduced toxicity [96]. 

A study comparing 3D-templated synthetic vascular graft material to a standard graft by using L-929 mouse fibroblast cells found no significant difference in cytotoxicity [97].

The overall search results indicated that synthetic and vascular graft materials can exhibit cytotoxicity, which appears to be influenced by factors like material composition and the cell types used for testing. Caution with these materials should be practiced while selecting.

Table 3 lists all possible types of synthetic biomaterials along with the general characteristics of their marketed products and manufacturer recommendations.

## 5. Barrier Membrane and Biomaterials

The concept of periodontal regeneration through membrane techniques is based on the idea that different tissues can be separated via the surgical insertion of barriers. The use of barrier membranes secures the defected area (for tissue regeneration) by preventing the invasion and occupation of epithelial cells, as soft tissue turnover occurs at a faster rate than bone and periodontal tissue creation [123]. When membranes and grafts are used together, the membranes serve to maintain, stabilize, and confine the graft materials, as well as reduce the rate of resorption. So far, the use of degradable and non-degradable barrier membranes has been established for the treatment of periodontal guided tissue regeneration (GTR) and guided bone regeneration (GBR) [124].

### 5.1. Biodegradable Barrier Membranes

Natural and synthetic polymer-based biodegradable membranes have several advantages, such as their low costs and low probability of second surgeries, and have the same treatment outcomes as non-biodegradable materials. However, biodegradable membranes are likely to be ranked as first-choice materials [125].

Despite all the advantages mentioned above, the resulting degradation byproducts, significant volume loss, infection, and residual virus in the case of applying animal-derived collagen and crosslinkers are likely to cause tissue regeneration failure [126].

In general, biodegradable membranes possess poor mechanical strength and are thus less efficient in space-making compared with non-biodegradable materials and mesh. Therefore, biodegradable membranes are used in combination with bone substitutes to maintain integrity and shape, and enhance the space-making quality of the system.

#### 5.1.1. Biodegradable Natural Polymers

The most commonly used natural polymers, such as type I and type III collagens, are derived from bovine and porcine [127].

Collagens are fundamental parts of connective tissues in the human body and are obtained from the intestine, tendons, and epidermis. They are later processed through decellularization, sterilization, and crosslinking, such as chemical treatment with genipin, carbodiimide, glutaraldehyde, and ultraviolet irradiation, to increase hydrolysis resistance. Crosslinking helps to improve mechanical strength, but chemical residues pose a threat of potential toxicity and inflammation [128].

Several studies have reported on the effectiveness of collagen membranes for bone augmentation and bone regeneration, and as a barrier in GBR, demonstrating bone formation potential almost equivalent to that documented with e-PTFE membranes. Additionally, it was stated that, beyond serving as a passive barrier, collagen membranes also aid in bone regeneration [129].

Compared with previous biodegradable membranes that facilitated bone regeneration, stromal cells attached to the collagen membrane stimulated the production of bone morphogenetic protein (BMP)-2 and basic fibroblast growth factor (FGF)-2.

In addition to collagen membranes, alginate and chitosan have also been used to prepare barrier membranes in dental research [130].

Chitosan membranes exhibit bacteriostatic and hemostatic properties with low mechanical strength, similarly to other natural polymers. Chitosan, in the form of fibers and films, has been used for artificial skin and surgical sutures because of its flexible workability, good biodegradability, and biocompatibility [131].

Similarly, alginate is a well-known natural polymer and impression material in dentistry with high biocompatibility [132].

However, in the case of chitosan and alginate barrier membranes, we found only a few documented in vivo investigations and no clinical trials with which to evaluate their efficacy in humans [130].

#### 5.1.2. Biodegradable Synthetic Polymers

Industrially manufactured reproducible aliphatic polyester barrier membranes are famous for their mechanical and biodegradable properties [133].

Several polymers, such as polyglycolic acid (PGA), poly(lactide-co-caprolactone) (PLCL), polylactic acid (PLA), polycaprolactone (PCL), and poly (lactic-co-glycolic acid) (PLGA), are available in the market. The ratio and type of polyester determine the quality and extent of the biodegradability of the aliphatic barrier membrane; for example, replacing PLA and PGA with PCL introduces the properties of low degradability and increased hydrophobicity to the barrier membrane, resulting in the extended lifespan of the membrane [134].

Furthermore, it is simple to impregnate active ingredients into the membrane that support tissue regeneration. These polyesters are used to treat minor defects in combination with other bone substitutes to improve their mechanical properties [135].

Bilayer membranes consisting of multiple polyesters such as PLGA and PLCL were developed with one porous layer and one solid layer to serve as a barrier and cell support system. During freeze-drying, the selected temperature determines the thickness of the formed layers. Although bilayer membranes have better operability, they possess lower mechanical strength. The PLGA bilayer membrane proved to have better bone regeneration and bone formation capacity compared with the monolayer membrane during in vivo experiments [136].

The same results were reported for the PLCL bilayer membrane, which had a comparatively slower degradation rate, actively preventing bacterial invasion and reducing bacterial adhesion to the membrane for GTR/GRR management [137].

To achieve improved bioactivity, mechanical strength, and the successful incorporation of active contents, polymeric membranes are usually manufactured with a combination of natural and synthetic polymers.

Another renowned synthetic biodegradable metallic membrane made of magnesium that degrades completely in the human body is the magnesium barrier membrane (NOVAMag^®^). It combines the advantages of non-resorbable and resorbable membranes, providing sufficient mechanical stability, space maintenance, and bioactivity while still being resorbable over a controlled period. Magnesium membranes have a resorption time of 8–16 weeks, providing sufficient time for bone regeneration while still being resorbable. This prevents the need for a second surgery to remove the membrane. Biomaterials were found to be safe and resorbable after healing. The resorbable fixation screws were also used to keep the membranes in place throughout the bone regeneration procedure [138].

### 5.2. Non-Biodegradable Barrier Membranes

Non-biodegradable barrier membranes, due to their advantage of having a controlled shielding period and showing an absence of material degradation byproduct issues, are frequently used for large-scale tissue regeneration. Additionally, they can be utilized in conjunction with small screws and metal pins to prevent the collapse of their morphology [139].

However, the main disadvantages associated with non-biodegradable membranes include the risk of complications during implantation, subsequent tissue regeneration, and later surgical removal [140].

Non-biodegradable membranes can be made of materials such as polytetrafluoroethylene (PTFE), expanded PTFE (e-PTFE), titanium meshes, polylactic acid, collagen, polyglycolic acid, and various polymers. Among these, e-PTFE was used to prepare the first documented barrier membrane [141].

PTFE is classified as a bioinert material because it is stable in vivo, biocompatible, and chemically stable, with an ability to withstand biodegradation and block the host’s immune system. Due to its high tissue barrier function, PTFE tends to lower blood flow and cause gingival dehiscence [142].

Furthermore, dense PTFE (d-PTFE), a more compact version of PTFE, has been introduced into the market and is currently under investigation to assess its effectiveness for GBR [129].

According to Ronda et al., while treating vertical bone deformities, d- and e-PTFE membranes showed almost similar clinical outcomes. However, the postoperative removal of the d-PTFE membrane was found to be easier and less disruptive to regenerated tissue compared with that of analogues of e-PTFE [143].

Recently, titanium-reinforced membranes have been used because of their potent space-making properties. Titanium, a bioinert material, is a pure metal with good mechanical strength, durability, biocompatibility, corrosion resistance, a low density, and the ability to form a passive layer [144].

Due to its possession of effective osseointegration properties, titanium directly connects to the bone via extracellular matrices in dental implants [145].

Hasegawa and colleagues prepared a regular hexagonal-shaped honeycomb structure within a titanium membrane and incorporated microperforations in constant intervals in the honeycomb sections. After the insertion of the autograft and prototype membrane into the bony defect sites, the results revealed the successful formation of osseous tissue within 26 weeks after implantation. In contrast, the presence of microperforations could have posed a challenge to the membrane retrieval procedure in this study [146].

Despite the space-making attributes of these nondegradable membranes, retrieval surgery can cause severe damage to regenerative tissues, increase susceptibility to infections, and hinder the healing process, posing significant challenges. 

The incorporation of inorganic materials into membranes not only promotes the regeneration of bones and tissues but also strengthens the system. The incorporation of β-TCP not only improved the mechanical strength of the membrane but also increased cell growth and cell adhesion by 50% [147]. The osteoconductivity and non-biodegradability of another inorganic biomaterial, hydroxyapatite, have been exploited to improve the outcomes of bone regenerative therapy [148]. That is why researchers have incorporated it into barrier membranes for guided regeneration treatment [149].

Furthermore, the osteoconductivity and biodegradability of the inorganic calcium phosphate material β-TCP were combined with those of PLGA and PCL to prepare a mesh membrane for guided regeneration purposes. The results of the in vitro and in vivo study confirmed improved proliferation, osteogenic differentiation, and bone formation even in the absence of bone substitutes [150].

Basile et al. prepared a composite membrane with hydroxyapatites and modified PCL. The results of the in vitro study reported osteogenic differentiation and favorable proliferation using mesenchymal stromal cells [151].

Furthermore, the incorporation of an enamel matrix derivative (EMD, fibroblast growth factors (FGF-2), BMP-2, -4, -7, and -12) has boosted the periodontal regeneration process [152].

### 5.3. Biomaterial Additive Membranes as Delivery Devices

PCL and PLA/cellulose acetate (CA) polymers were combined to create electrospun nanofiber membranes. To enhance antibacterial activity, bone conductivity, and bone binding ability, scientists incorporated green-synthesized silver nanoparticles and hydroxyapatite nanoparticles, resulting in a promising GBR fiber membrane. The antibacterial effect was observed for over a month with a 40 mm inhibition zone. Furthermore, research investigations have focused on designing and preparing GBR membranes with a bilayer structure, demonstrating enhanced GBR capabilities through the blending of multiple components [153].

Semi-synthetic tetracyclines are recommended as beneficial supplements for treating periodontal disease. In vivo evaluations were performed by adding 25% doxycycline to a polymeric GTR membrane made of polyglycolic acid and polylactic acid, which resulted in improved osteogenesis for repairing periodontal bone in dogs [154].

Veríssimo and colleagues formulated membranes with hydroxyapatite and collagen for curing calvaria defects, showing exceptional results in defect healing but compromised biodegradability [155].

Amorphous and biodegradable bioactive glass is generally made of silicon dioxide and easily releases silicate and calcium ions, which aid in bone binding and increase osteoblast activity [156]. That is why the addition of bioactive glass in biodegradable barrier membranes helps stimulate mineral deposition and osteoblastic cell activities. This combination has been proposed as effective alongside collagen membranes and the FGF-2 solution for bone repair in rat calvaria defects [157].

The specific cytotoxicity may vary depending on the membrane composition and manufacturing process. Most barrier membranes manufactured with synthetic polymers are considered non-cytotoxic and biodegradable. According to research data, collagen-based resorbable barrier membranes, such as Bio-Gide^®^, RESODONT^®^, and GENTA-FOIL resorb^®^, were evaluated for cytotoxicity. GENTA-FOIL resorb^®^ was found to be toxic while the other two membranes showed a higher rate of cell viability and proliferation [158].

The cytotoxicity of chitosan-based barrier membranes is correlated with the molecular weight; higher molecular weights are considered to be responsible for increased inflammatory responses. The barrier membranes commonly used for GBR meet the requirements for the cytotoxicity of surgical implant materials. Non-absorbable barrier membranes like ePTFE have lower cytotoxicity in comparison to absorbable membranes (collagen and polylactic-co-glycolic acid); however, PLGA and collagen are still considered biosafe [159]. Synthetic polymers and collagen-based membranes generally demonstrate acceptable cytotoxicity profiles that are suitable for clinical use in guided bone regeneration procedures.

The use of barrier membranes in dentistry has grown with the advancements in polymer sciences. Membranes for GTR and GBR are expected to have several properties that collectively enhance their quality. Current research is focused on investigating the functionality of barrier membranes to promote tissue regeneration activities, emphasizing the need for further study into the interactions and degradation dynamics of growth factors and antibacterial agents loaded into these membranes. Table 4 presents an overview of available biomaterials used in manufacturing barrier membranes, outlining their advantages, disadvantages, and marketed products to provide a piece of concise information to the readers.

Currently, no standards or criteria have been established for selecting barrier membranes for guided tissue and bone regeneration. The only way to select an appropriate membrane for personalized use depends on factors such as bone replacement types, growth hormones, and membrane properties.

## 6. Outlook

Professionals in medicine and dentistry are using synthetic materials more frequently because of their self-hardening, ease of handling, and injectability. Additional benefits of these materials are their large-scale, controlled, and planned manufacturing.

### 6.1. Treatment Plan Considerations by Dentists

Several variables, including the qualities of the graft material, the patient’s health treatment objectives, and the location of the surgical site, are deciding factors for suggesting a suitable treatment plan. The optimal bone graft material should encourage the formation of new tissues by seamlessly blending with the patient’s original bone and minimizing the chance of rejection at the surgical site.

For dental operations, autografts are usually taken from the chin, hard palate, mandible ramus, or jaw. The tissue graft may come from the hip or shinbone if there is not enough bone available in the aforementioned locations. Since bone is a natural part of the patient’s body, the primary advantage of an autograft is the decreased probability of graft rejection. However, it is undeniable that unpredictable and rapid resorption and the need for additional surgeries to obtain grafts are associated drawbacks [171].

Typically, allografts are sourced from cadaveric donors. Before any bone or other tissue is utilized, the donor must undergo a careful examination to ensure the absence of infectious conditions. To minimize any possible immunological reactions and ensure compatibility with the recipient, the harvested bone undergoes a number of treatments. These may include irradiation (radiation exposure), freeze-drying, decellularization, sterilization, and the use of other chemicals such as hydrochloric acid. The primary disadvantages are the risk of an immunological response, tissue rejection, and reduced osteoconductivity due to the removal of growth factors during manufacturing processes [172]. 

An animal source, usually a cow or pig, is used to obtain a xenograft. When this type of graft is used, the bone is meticulously prepared through hydrothermal, chemical, and hydrazine treatments, resulting in the majority of the remaining tissue being composed of mineral elements. One benefit of using xenografts is the ease of obtaining large bone samples with the appropriate microstructure, which improves compatibility at the planned surgical location. Xenografts serve as both a biological and mechanical placeholder in the jaw, effectively aiding in bone reconstruction. Initially, the xenograft provides physical support to the surgery site, and over time, the body gradually replaces the xenograft with newly formed bone [173].

Alloplastic transplants are not derived from human or animal sources. Grafts made of alloplastic materials can come from synthetic materials, natural materials, or a mixture of both. They are preferred by many dentists as they are non-allergenic and eliminate the need for tissue extraction and incorporation from internal or external sources [20].

Because of its improved mechanical strength, longevity, and good bone integration, hydroxyapatite has been amongst the most commonly used materials. As a matter of fact, hydroxyapatite takes up a significant part of human bone [174]. However, it is losing its popularity in dentistry due to its poor resorbability, stability, and susceptibility to bone fracture.

Ceramics, and nonmetallic and inorganic biomaterials can tolerate extremely high temperatures and are incredibly hard. The main advantages of ceramic grafts are their capacity to fuse with native bone, encouraging the formation of new bone, and their ease of storage [37].

Similarly to ceramics, bioglass, also known as bioactive glass, is an additional viable material for dental bone grafts. Bioglass can adhere smoothly and fully to bone upon exposure to body fluids, allowing it to perfectly mold into a jawbone socket [21]. Unlike certain other types of bone graft, it comes in a variety of pliable forms, such as pastes and putties. Polymer-based alloplastic grafts, whether synthetic or natural, are also a popular option among dentists since they fully resorb in the body over time [20].

A unique type of transplant graft that deserves mention is the sinus lift. This procedure involves raising the sinus floor for a dental implant and filling the space below with packing material [175]. The sinus lift procedure can be combined with grafts to obtain better results in therapy.

Bone grafts are often used in combination with biocompatible membranes to prevent tissue growth that may interfere with bone growth, where the membranes act as space holders and stabilize the blood clot, which could otherwise interfere with healing [176].

### 6.2. Future Considerations

The development of superior or innovative substitutes for bone grafts is one of the primary objectives of biomedical research. For example, the delivery of medications or antibiotics in the context of treating infections and imitating the structure of bones is being explored through three-dimensional printing. Therefore, composites made of minerals and ceramics have become widely popular [177]. There have also been attempts to incorporate growth factors and osteogenic cells into these structures to alleviate bone deficiencies associated with poor vascularity.

Nanotechnology can be used to generate more bioactive bone replacements that inhibit osteolytic processes, initiate reparative cascades, or improve the biological performance of cells. The most significant impacts on the synthesis of new bone were found in trials involving mesenchymal cells and growth factors. 

The porosity and particle size of synthetic graft substitutes are the main parameters for evaluating bone graft ingrowth efficacy. Researchers fabricated bioceramics and biopolymers nanocomposites (HA-fucoidan) to assess its mineralization effect in a rabbit bone defect model that mimics the bone ultrastructure. The results confirmed that these materials are suitable for bone replacement therapy, as they induce fibroblast growth factor-2, promote angiogenesis, and stimulate collagen production [178].

Similarly, using silk from the silkworm *Bombyx mori* as a scaffold is another intriguing research field. Myel et al. [179] used biodegradable silk implants to demonstrate the efficacy of porous silk fibroin scaffolds in reconstructing mouse calvarial defects by inducing bone formation within 5 weeks. Moreover, a study by Pina et al. demonstrated the effectiveness of treatment when ionic-doped calcium phosphates were deposited into a silk scaffold to promote bone regeneration [180].

Developing a porous structure that is mechanically resilient and capable of effective osseointegration and vascularization has been identified as the main issue in material development. Unfortunately, synthetic bone substitutes are only osteoconductive, with bone growth restricted to the outer layer [181]. This underscores the need for the development of meticulous design with innovative materials, considering crucial biological properties, such as pore size, density, morphology, interconnectivity, and resorbability [17].

Furthermore, the incorporation of various growth factors, including EMD (enamel matrix derivative), rhPDGF (recombinant human platelet-derived growth factor-BB), PRF (platelet-rich fibrin), and FGF (fibroblast growth factor) can encourage periodontal regeneration. The application of rhBMP-2 (recombinant human bone morphogenetic protein-2) has demonstrated promising outcomes related to extra bone formation. However, combining rhBMP-2 with synthetic biphasic calcium phosphate scaffolds can further improve its osteoinductive qualities and promote new bone regeneration [182]. Additional studies should be conducted on certain growth factors for periodontal bone regeneration.

In tissue engineering, although scaffolds are a vital component of newly developed strategies, their implantation at the sites might cause inflammatory reactions and hinder integration and regenerative outcomes. This limitation has prompted scientists to find or develop biomaterials with immunomodulatory qualities. Pro-resolving lipid mediators (lipoxin, resolvins) are interesting candidates for periodontal tissue therapy approaches due to their supportive role in both tissue regeneration and inflammation resolution, as confirmed by preclinical studies. Validating the regenerative effects of pro-resolving lipid mediators requires further clinical investigations [183].

Moreover, the future of dental implants and bone grafting is being shaped by the development of hybrid grafts loaded with growth factors and/or living osteogenic cells to induce bone regeneration. Bone substitutes that release platelet-derived growth factors or bone morphogenic proteins under controlled conditions are good examples for clinical use. The use of osteogenic and angiogenic hydrogels as bone graft technology is currently under investigation. Recently, osteogenic polypeptide hydrogel has been combined with photo-crosslinked peptides to promote the healing and long-term regeneration of defective bone [184].

Furthermore, fast-resorbing polymers exhibit porous structures, which could be a characteristic beneficial to achieving better tissue growth and vascularization in bone graft therapy [185].

However, the authenticity of the information available regarding these newer materials may be questioned because the data mainly originate from different case studies or experimental clinical animal models. To ascertain the clinical viability and benefits of each substance and to introduce more economically viable products, it would be imperative to conduct more standardized preclinical and clinical trials.

Regarding guided non-absorbable and absorbable synthetic polymer membranes, one limitation is poor biocompatibility. For natural collagen membranes, rapid degradation, poor mechanical strength, and lack of proper barrier function when applied to large defect wounds are major challenges. Moreover, most barrier membranes are not bioactive and therefore do not help actively promote periodontal tissue regeneration.

Extended crosslinking may prolong the degradation time, but could impede tissue integration or initiate a foreign body reaction.

Therefore, to improve membrane biocompatibility, the development of functional gradient membranes, nanofiber scaffolds, and electrospinning (ELS) techniques is a potential solution. Furthermore, the addition of metal alloys (such as magnesium and zinc), the concentration of nanoparticles, and the use of a directional membrane fiber arrangement are employed to control the fiber diameter and porosity of the membrane and to improve its barrier function. The mixing of natural and synthetic polymers with other biomaterials that have varying degradation rates can alter the degradation rate while maintaining barrier function. Furthermore, the use of 3D printing technology to improve mechanical strength can be considered a potential solution [186].

**Figure 1:**
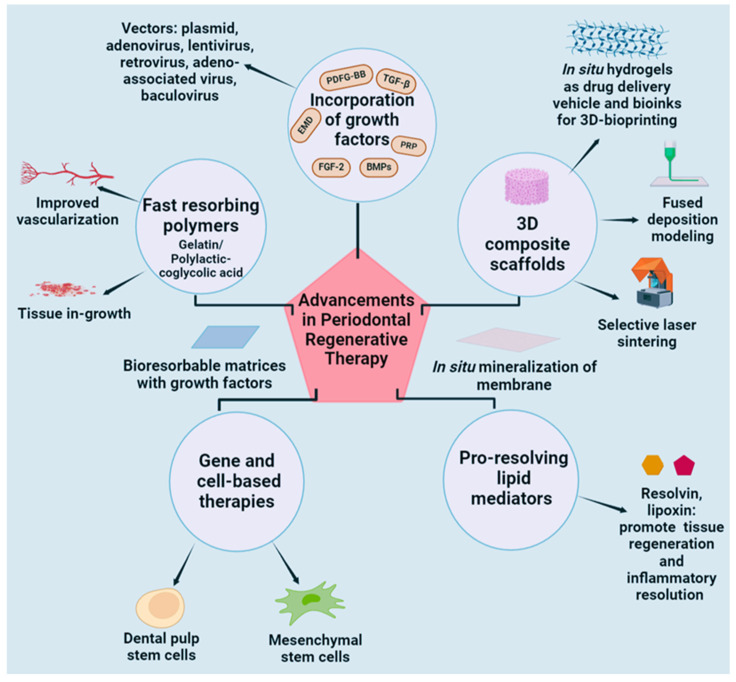
Future prospects and advancements in periodontal regeneration therapy.

Hybrid membranes present a solution to resorption and achieving stability by combining a resorbable matrix with a non-resorbable surface. This combination permits the non-resorbable component to offer initial stability and space maintenance while permitting the resorbable component to gradually resorb and integrate. Figure 1 summarizes all possible approaches to improve treatment options with desirable results.

To provide a solid foundation for future implant placement and ideal esthetics in clinical settings, socket preservation treatments aim to prevent the loss of alveolar ridge dimensions right after tooth extraction. This involves reducing bone resorption, maintaining the gingival contour, and preserving ridge dimensions by placing immediate bone grafts and barrier membranes in the extraction socket after tooth removal [187].

## 7. Conclusions

Developing high-quality products involves combining various alloplastic bone grafting materials. Furthermore, integrating recombinant DNA technology allows for the incorporation of one or more growth factors with alloplastic grafts to promote the formation of new bone.

Despite the advancements highlighted in this review article, dental biomaterials that possess a porous structure, controlled degradation, mechanical stability, and remodeling ability at a pace equivalent to that of new bone production still require further development. Further comparative studies on humans may shed light on whether or not these advancements in synthetic bone substitutes will lead to better bone remodeling and regeneration, both in terms of quantity and quality, compared with natural biomaterials.

Customized treatment plans depend on the needs of each patient and clinical circumstances, including bone quality, cost, and surgical expertise. Concisely, the potential to improve the effectiveness and consistency of bone grafting and membrane procedures in periodontal regeneration can be achieved through the development of advanced biomaterials, tissue engineering strategies, 3D scaffolds that mimic the exact picture of defective sites, and grafting combined with stem cell treatment options. Future developments in these fields are expected to enhance existing periodontal therapy strategies, leading to improved patient care and compliance.

## Figures and Tables

**Table 2:** Natural and synthetic graft materials available in commercial tissue banks and markets along with general characteristics.

Graft Type	Merits	Demerits	Mechanism and Level				Market Products	Ref.
			Osteoconduction	Osteoinduction	Osteogenesis	Osteointegration		
Autograft The gold standard for bone grafting, but only a limited amount of graft material is available. Unpredictable and rapid resorption are possible drawbacks. The additional surgical site increases the risk of infection [21].								
Cancellous	Revascularization due to a large surface area.	Poor mechanical strength.	Present (high)	Present (high)	Present (high)	Present (high)		[33]
Cortical	Provides structural support and mechanical stability.	Takes longer to remodel than a cancellous graft.	Present (low)	Present (low)	Present (low)	Present (low)	-	[21]
Vascularized cortical	Provides structural support and rapid healing, osteoprogenitors, osteocytes, and cells preserved in the graft.	Implantation and harvesting are challenging.	Present (medium)	Present (low)	Present (medium)	-	-	[34]
Bone marrow aspirate	Harvesting is minimally invasive.	Presence of fewer stem cells in the graft.	Present (low)	Present (medium)	Present (high)	-	-	[35]
Platelet-rich plasma (PRP)	Easy to acquire, affordable, elicits the migration of MSCs to target site, smaller amounts of harvested bone needed.	Complexity of the procedure, variation in preparation method.	-	Present (high)	-	-	-	[36]
Allografts Second surgical site is avoided with no additional suffering or pain, minimal risk of infection, and unfavorable immunological response [37].								
Cancellous	Low residual moisture due to freeze-drying, shelf-life of 4–5 years.	Mechanically weak grafts, difficulty to incorporate as grafts in fibrous tissue are incorporated by the body, immunogenicity.	Present (high)	Present (with fresh allografts)	-	Present (medium)	Cancellous chips/freeze-dried (Musculoskeletal Transplant Foundation: Edison, NJ, USA), Puros^®^ (Zimmer Biomet: Warsaw, IN, USA), Raptos^®^ (Citagenix: Laval, QC, Canada)	[38]
Cortical	Mechanically strong and can support the load.	Inflammatory response delays healing, immunogenicity.	Present (high)	Present (with fresh allografts)	-	Present (low)	Raptos^®^ (Citagenix: Laval, QC, Canada)	[39]
Demineralized bone matrix (DBM)	Holds growth factors, low immunogenicity.	Variations in the content of growth factors amongst different batches, poor mechanical strength.	Present (low)	Present (low)	-	Present (medium)	Grafton^®^ (BioHorizons: Birmingham, AL, USA), Opteform^®^ (Exatech Inc.: Gainesville, FL, USA), OsteoSponge (Xtant medical: Belgrade, MT, USA), DynaBlast^®^ (Keystone Dental Group: Burlington, MA, USA)	[40]
Xenografts Good mechanical strength, ample supply, and low degradation rate [41].								
Xenografts	Similarity to human bone a calcium/phosphate ratio of 1.67 identical to human bone composition, low immunogenicity, and biomechanical properties same as the bone.	Ethical and religious issues, risks of disease transmission.	Present (high)	Present (low)	-	Present (low)	Gen-Os^®^ (Cortico-cancellous heterologous bone mix) (Tecnoss dental, Torino, Italy), Cerabone^®^ (Bovine cancellous bone grafting) (Straumann: Basel, Switzerland), Straumann^®^ XenoFlex (biphasic composition: collagen and hydroxyapatite (xenogenic), (Straumann: Basel, Switzerland), Bio-Gen (equine bone) (Bioteck: Arcugnano, Vicenza (VI), Italy), InterOss^®^ (bovine bone) (Sigmagraft Inc. biomaterials: Fullerton, CA, USA), NuOss (bovine bone) (ACE Surgical Supply Company, Inc: Brockton, MA, USA)	[42]
Alloplastic Various options for graft materials, custom scaffolds (3D printed, injectable), lack of growth factors [43].								
Calcium phosphate ceramics/ biphasic calcium phosphate ceramics	Similar composition to bone, has excellent intraparticle cohesivity, resorbability of 85% β-TCP, and osteoconduction of 15% hydroxyapatite.	Compromised mechanical strength, difficult to mold.	Present (medium)	-	-	Present (medium)	Teebone^®^ (Medibrex: Zalqa, Lebanon), Mastergraft™ (Medtronic Sofamor Danek, Memphis, TN, USA), Ossfinity^®^ (Leader Biomedical: Amsterdam, The Netherlands), Maxresorb^®^ (Botiss biomaterials GmbH: Zossen, Germany)	[44,45]
Beta tricalcium phosphate	Bears the most similarity in composition to bone and is called the ‘gold standard’ of synthetic grafts.	Unpredictable degradation, rapid resorption.	Present (low)	-	-	-	Vitoss^®^ (Stryker Corporation: Kalamazoo, MI, USA)	[46]
Hydroxyapatite	Biocompatible and comparatively possessing high tensile strength.	Slow resorption.	Present (low)	-	-	-	Collagraft^®^ (Zimmer Biomet: Warsaw, IN, USA), Healos^®^ (DePuy Synthes Spine: Raynham, MA, USA)	[47]
Calcium phosphate cement	Easy to mold.	Poor mechanical strength.	Present (medium)	-	-	-	CopiOs^®^ (ZimVie: Westminster, CO, USA), Norian^TM^ (Synthes GmbH: Eimattstrasse, Oberdorf, Switzerland), ChronOS inject^TM^ (DePuy Synthes Spine: Raynham, MA, USA), Hydroset^TM^ (Stryker Corporation: Kalamazoo, MI, USA)	[48]
Calcium sulfate	High biocompatibility, self-setting strength, and resorption with minimal inflammation, low cost, easy to prepare.	Risks of wound drainage, poor mechanical strength. Resorbs faster than the dissolution of bone itself.	Present (low)	-	-	Present (medium)	OsteoSet^®^ (Wright Medical Group: Memphis, TN, USA)	[49]
Bioactive glass	Possesses antibacterial activity, formation of bonds between tissues and bones, stimulates osteogenesis, good biocompatibility	Low toughness and mechanical strength	Present (low)	-	-	-	BioGran^®^ (Biomet 3i Innovations Inc.: Plam beach gardens, FL, USA)	[50]
PMMA bone cement	Secures implants in place, treats bone defects.	Poor adhesion, heat sensitivity, and the reaction to such a foreign body can result in a loosening of the implant and a risk of bone cement implantation disorder.	-	-	-	-	C-ment^®^ (Leader Biomedical: Amsterdam, The Netherlands)	[51]
BMP-2, BMP-7	Better bone regeneration reported in smokers.	Off-label use, swelling issues, risk of ectopic bone formation.	Present (with collagen carriers)	Present (low)	Present (low)	-	BMP-7/OP-1 (United States Biologicals: Swampscott, MA, USA), Infuse^TM^ Bone Graft (Medtronic: Dublin, Ireland)	[52]
Recombinant human platelet-derived growth factor-BB homodimer (rhPDGF-BB) and ß-TCP	Less painful procedure as compared to natural grafts, treats intrabony, furcation periodontal defects.	Expensive, may require additional surgery, swelling, pain, bleeding.	Present (high)	-	-	-	GEM 21S^®^ (Geistlich Pharma, Wolhusen, Switzerland)	[53]
iFactor (P-15)	Outcomes like in the case of autografts.	Dysphagia post-treatment.	Present (in combination with other synthetic grafts)	-	-	-	i-FACTOR Flex bone graft, (Cerapedics: Broomfield, CO, USA), p-15™ (Cerapedics: Broomfield, CO, USA)	[54]
Polymers (chitosan)	Present low immunological rejection.	Poor resorption.	Present (low)	-	-	-	Commercially provided by manufacturers	[55]

**Table 3:** Synthetic biomaterial products along with general features and recommendations from manufacturers.

Synthetic Biomaterials	Features Related to the Product	Available Brand	Available Form	Manufacturers	Comments by Manufacturers	Ref.
Bioactive glass	Osteostimulation, osteoconduction, anti-inflammatory, and antibacterial activities (local, transient)	PerioGlas^®^	Cups, syringes	NovaBone (Jacksonville, FL, USA)	When the material is implanted in living cells, its surface undergoes a time-dependent kinetic alteration.	[98]
BCP (60% HA/40% β-TCP)	100% degradable and bioresobable.	Guidor^®^ easy-graft	Injectable/sterile powder	Sunstar (Osaka, Japan)	Repairs and fills the defects left by apicoectomy, autologous bone, and cyst removal.	[99]
Porous β-TCP	Osteoconduction, osteoinduction, resembling human cancellous bone.	Vitoss^®^	Moldable packs, malleable strips, and morsels.	Stryker Corporation (Kalamazoo, MI, USA)	When mixed or hydrated with bone marrow aspirate (BMA), it exhibits the same components of bone healing as the gold standard (iliac crest bone graft).	[100]
Phosphocalcium Cement (a mixture of calcium phosphate salts) Liquid phase: solution of Na_2_HPO_4_ (pH 8.7) to accelerate bone substitution setup	Osteoconduction and surface osseointegration, slow resorption.	Eurobone^®^ 2std	Injectable synthetic cement	FH Ortho (Mulhouse, France)	Within 24 h of implantation, it is able to achieve maximum strength.	[101]
β-TCP	Osteoconduction, osteointegration, and biocompatible improved mechanical strength.	HydroSet XT	Injectable	Stryker Corporation (Kalamazoo, MI, USA)	Hydroset offers rigidity to encourage new bone formation.	[102]
Silicated β-TCP	Resorbable, mechanically stable, minimizing micro-movement.	IngeniOs^®^	Particles 1–2 mm	Zimmer Biomet (Warsaw, IN, USA)	Combination with biologic drivers in autologous PRP, bone marrow, or stem cells.	[103]
HA	Biocompatible, osteoconduction, non-immunogenicity.	B-OstIN^®^	Granules, rods, and blocks	Basic Healthcare (Himachal Pradesh, India)	The most active component among ceramics, non-toxic, promotes bone fusion within three months with no known side effects.	[104]
Bioactive glass	Highly resorbable due to small size.	BioGran^®^	Granules	Biomet 3i Innovations Inc. (Plam beach gardens, FL, USA)	Offers the special environment of protection required for osteogenesis.	[105]
60% HA, 40% (β-TCP)	Reproducibility, biocompatibility, and osteoconduction facilitate osteoblast migration and vascularization.	BoneCeramic™	Granules	Straumann (Basel, Switzerland)	The maximum space for the formation of new bone is provided by minimal amounts of a highly porous substance.	[106]
nano-HA	Osteoconduction, partially resorbable.	Fisiograft bone granular	Granules 250–1000 microns	Ghimas (Bologna, Italy)	Strongly interconnected porosity.	[107]
Calcium phosphate, lyophilized type I bovine collagen	Identical to human cancellous bone, promotes bone regrowth, resorbable.	CopiOs^®^	Paste, sponge	ZimVie (Westminster, CO, USA)	Enhance the solubility of bone morphogenetic proteins (BMPs) and other osteoinductive growth factors, a second surgical harvest procedure is not required.	[108]
Coralline HA	Similar to human cancellous bone, resorbable.	Pro Osteon^®^	Granules	Zimmer Biomet (Warsaw, IN, USA)	Residual material stays for years in ceramic form.	[109]
Calcium sulfate, calcium phosphate, demineralized bovine bone	Osteoconduction, osteoinduction, bioresorbable.	Pro-Stim^®^	Injectable inductive graft	Wright Medical Group (Memphis, TN, USA)	Do not supplement the graft with other drugs or substances; using additional chemicals or solutions may change the safety profile of the mixture. New bone formation occurs in 13 and 26 weeks.	[49]
β-TCP (>99%)	Resorbable, non-allergenic, and without systemic toxicity.	Cerasorb^®^	Granules (size: 150–2000 microns)	Curasan (Frankfurt am Main, Germany)	Promotes bone formation due to sufficient space among particles.	[110]
Porous HA	Long-lasting osseous integration, osteoconduction	ENGIpore^®^	-	Finceramica (Faenza RA, Italy)	Able to withstand the compressive forces associated with real bone.	[111]
β-TCP +HA	Osteoconduction, resorbable.	Calciresorb^®^ C35	Granules	Ceraver (Roissy Cdg Cedex - France)	-	[112]
Silicate-substituted calcium phosphate	Osteoconduction, osteostimulation, no risk of disease transmission, accelerated bone growth.	Actifuse^®^ ABX	Granules	Baxter (Deerfield, IL, USA)	Bony regeneration may be hampered by a patient’s metabolism.	[113]
Calcium sulfate, antibiotics	Resorbable	Stimulan^®^	Beads	Biocomposites Ltd. (Keele, UK)	Can be mixed with tobramycin. vancomycin, gentamicin, it resorbs faster than bone.	[114]
60% HA, 40% β-TCP	Structurally and chemically resembles and mimics cancellous bone, resorbable, osteoconductive, 70% porous	OpteMx^®^	Particles, sticks, wedges, cylinders of various sizes	Exatech (Gainesville, FL, USA)	Quite high compressive strength (2.6 MPa). It is advised to use rigid fixation techniques until OpteMx is reabsorbed.	[115]
85% β-TCP, 15% HA	Osteoconductive, resorbable	Mastergraft^®^	Granules, putty	Medtronic (Dublin, Ireland)	Mastergraft is offered as separate grains or in combination with collagen to produce a putty-like substance with cohesive and moldable properties.	[116]
Calcium phosphate and polymers	Biocompatible, moldable, and resorbable due to the addition of polylactide/glycolide copolymer fibers.	Norian Drillable Bone Void Filler	Injectable, sterile powder	Synthes GmbH (Eimattstrasse, Oberdorf, Switzerland)	Achieves ideal bone defect filling and a compressive strength of 35 MPa in 24 h.	[117]
Biphasic calcium phosphate	Rapidly absorbed; nano-HA particles provide an extremely large surface area for cellular interactions	Maxresorb^®^	Injectable paste, granules	Botiss biomaterials GmbH (Zossen, Germany)	100% synthetic, almost the risk of infection, ensures a high degree of reproducibility and material safety.	[76]
β-TCP	Osteoconductive, resorbable, and mimics the structure of cancellous bone.	Cellplex^®^ TCP	Granules	Wright Medical Group (Memphis, TN, USA)	Highly porous, interconnected structure, excellent carrier of BMA, packed in Marrow Infusion Chamber INFILTRATE^®^ to give surgeons an easy way to combine BMA with CELLPLEX^®^ TCP.	[118]
Calcium sulfate	Resorbable	MIIG^®^ X3	Injectable, paste	Wright Medical Group (Memphis, TN, USA)	It is essentially the same as other available bone void fillers.	[46]
Calcium sulfate, Calcium phosphate, β-TCP	Slow-resorbing, mechanically strong, and more dense bone	ProDense^®^	Injectable	Wright Medical Group (Memphis, TN, USA)	Regenerates new bone that bears nearly six times more compressive strength than autograft bone.	[119]
Calcium sulfate	Osteoconduction, resorbable	OsteoSet^®^	Beads	Wright Medical Group (Memphis, TN, USA)	Works as a passive osteoconductive scaffold.	[120]
β-TCP (80), type I bovine collagen (20)	Osteoconduction	Integra Mozaik^TM^	Putty	Integra LifeSciences Corp. (Princeton, NJ, USA)	Mimics the original composition of bone, bends to fit uneven surfaces, and retains bioactive fluids within the scaffold to aid in protein binding.	[121]
Synthetic HA	Osteoconduction, biologically safe and biocompatible	Apaceram^®^	Block, granules	HOYA Technosurgical Corporation (Shinjuku-ku, Tokyo, Japan)	Available in different porosity ranges and mechanical strengths for intended usage.	[122]

**Table 4:** Biomaterials for barrier membranes, with their advantages, disadvantages, and marketed products.

Biomaterials for Barrier Membrane	Resorbable/Non-Resorbable	Advantages	Disadvantages	Commercial Products	Ref.
Non-biodegradable					
Metal: Titanium, titanium alloy	Non-resorbable	Improved mechanical strength, stability, durability, high biocompatibility, and barrier function	Expensive second surgery is required for membrane removal	GDT Titanium Mesh Membranes (GDT dental implant: Beer Sheva Israel), OSS Builder (Osstem Implant: Auckland, New Zealand), Titanium Mesh (Stanford Advance Materials: Lake Forest, CA, USA)	[146,160]
Cobalt, cobalt alloy	Non-resorbable	Affordable price, improved space-making, and mechanical strength	Less biocompatible	n!ce^®^ Cobalt-Chromium (Straumann: Basel, Switzerland)	[161,162]
Polytetrafluoroethylene (PTFE) expanded PTFE (e-PTFE)	Non-resorbable	High biocompatibility and stability, stiffness and space maintainer, bacterial resistance	Membrane exposure, second surgery is required	Gore-Tex^®^ (W. L. Gore & Associates: Newark, DE, USA), Cytoflex^®^ (Unicare Biomedical: Laguna Hills, CA, USA), Cytoflex^®^Tef-Guard^®^ (Unicare Biomedical: Laguna Hills, CA, USA)	[163,164,165]
Titanium-reinforced polytetrafluoroethylene (PTFE)	Non-resorbable	Hard tissue reconstruction, both vertically and horizontally, remains stable with minimal membrane exposure.	Ridge augmentation and additional surgeries are required.	Gore-Tex-TI (GORE-TEX: Newark, DE, USA), Cytoplast^TM^ Ti-Enforced^®^ ePTFE ^®^ (BioHorizons, Birmingham, AL, USA), NeoGen^®^ Ti-reinforced (Straumann: Basel, Switzerland), OsseoGuard^®^ PTFE (ZimVie: Westminster, CO, USA), Cytoflex^®^ Ti-reinforced (Unicare Biomedical: Laguna Hills, CA, USA)	[166]
High-density PTFE (d-PTFE)	Non-resorbable	Improved bacterial resistance, prevents infections, highly stable, and offers better intracellular penetration due to pore size.	Chances for second surgery, has lower porosity compared with PTFE, not a fully inert material.	Cytoplast^®^TXT-200 ^®^ (BioHorizons, Birmingham, AL, USA), NeoGen^®^ (Neoss: Zurich, Switzerland), Permamem^®^ (Straumann: Basel, Switzerland), OsseoGuard^®^ (Zimmer Biomet: Warsaw, IN, USA)	[142]
Biodegradable (natural polymers)					
Alginate, Chitosan, Agarose	Resorbable	Prevents the need for surgical removal of the membrane and is highly biocompatible.	Suspicious barrier function, few documented trials are available, rapid resorption with weak mechanical strength.	-	[130,149]
Collagen	Resorbable	Avoids surgical removal, is biocompatible and suitable for wound healing and barrier functions.	Risk of disease transmission, uncontrolled biodegradability, and low mechanical strength	Cytoplast^®^ RTM collagen (Osteogenics: Lubbock, TX, USA), AlloDerm^®^ SELECT^TM^ RTM (BioHorizons, Birmingham, AL, USA), BioMend^®^ and BioMend Extend^TM^ (ZimVie: Westminster, CO, USA), Ossix Plus^®^(Dentsply Sirona Inc, Charlotte, NC, USA), Geistlich Bio-Gid^®^ (Geistlich Pharma, Wolhusen, Switzerland)	[128,167]
Biodegradable Synthetic Polymer					
Aliphatic polyesters (PLA, PGA, and PCL), and copolymers	Resorbable	Biocompatible and reproducible, with a controlled mechanism and improved barrier functionality.	Mechanically weak, produces cytotoxic byproducts	Resolut Adapt^®^ (W. L. Gore & Associates: Newark, DE, USA), Vicryl periodontal mesh (Ethicon, Inc: Raritan, NJ, USA), Atrisorb^®^ (TOLMAR Inc: Fort Collins, CO, USA)	[123,136,168,169]
Cellulose acetate	Resorbable	Inert, biocompatible, cost-effective, stable; its durable renewability exhibits higher chlorine resistance.	Unstable at elevated temperatures, very thin and asymmetric structure, biodegradable, prone to rejection with acid or basic hydrolysis.	Millipore^®^ (Merck KGaA, Darmstadt, Germany)	[170]

## Data Availability

Not applicable.
